# Identification and Prediction of Tuberculosis in Eastern China: Analyses from 10-year Population-based Notification Data in Zhejiang Province, China

**DOI:** 10.1038/s41598-020-64387-5

**Published:** 2020-05-04

**Authors:** Kui Liu, Tao Li, Avina Vongpradith, Fei Wang, Ying Peng, Wei Wang, Chengliang Chai, Songhua Chen, Yu Zhang, Lin Zhou, Xinyi Chen, Qiao Bian, Bin Chen, Xiaomeng Wang, Jianmin Jiang

**Affiliations:** 1 Department of Tuberculosis Control and Prevention, Zhejiang Provincial Center for Disease Control and Prevention, Hangzhou, Zhejiang Province People’s Republic of China; 2 Key Laboratory of Vaccine, Prevention and Control of Infectious Disease of Zhejiang Province, Zhejiang Provincial Center for Disease Control and Prevention, Hangzhou, Zhejiang Province People’s Republic of China; 3 Chinese Center for Disease Control and Prevention, Beijing, People’s Republic of China; 4 Institute for Health Metrics and Evaluation, University of Washington, Seattle, Washington United States of America; 5 Ningbo University, Ningbo, Zhejiang Province People’s Republic of China

**Keywords:** Diseases, Health care, Medical research

## Abstract

Tuberculosis, a severe infectious disease caused by the Mycobacterium tuberculosis, arouses huge concerns globally. In this study, a total of 331,594 TB cases in Zhejiang Province were notified during the period of 2009–2018 with the gender ratio of male to female 2.16:1. The notified TB incidences demonstrated a continuously declining trend from 75.38/100,000 to 52.25/100,000. Seasonally, the notified TB cases presented as low in January and February closely followed an apparent rise in March and April. Further stratification analysis by both genders demonstrated the double peak phenomenon in the younger population (“15–35”) and the elders (“>55”) of the whole group. Results from the rate difference (RD) analysis showed that the rising TB incidence mainly presented in the young group of “15–20” and elder group of “65–70”, implying that some implementations such as the increased frequency of checkup in specific student groups and strengthening of elder health examination could be explored and integrated into available health policy. Finally, the SARIMA (2,0,2) (0,1,1)12 was determined as the optimal prediction model, which could be used in the further prediction of TB in Zhejiang Province.

## Introduction

Tuberculosis (TB), as a severe infectious disease, is still a leading cause of death worldwide, causing massive concern in public health continuously^1^. Although new diagnosis technology, novel drugs for treatment, relevant support service, and positive political commitment were promoted steadily in recent years, there are still more than 10 million newly diagnosed patients reported globally, and 5% of them were identified as rifampicin-resistant cases^2^. More seriously, based on the WHO’s release, only 64% of cases were diagnosed and recorded formally, an estimated 1.3 million TB cases resulted in death among HIV-positive people, and an additional 0.3 million deaths resulted among HIV-negative people in 2017^1^. The majority of infected cases (87%) are found in 30 high-burden countries. China ranked as second place among high-burden countries, contributing to 9% of the estimated global totals. Additionally, according to a release by the World Health Organization (WHO) in 2018, drug-resistant TB, including resistance to rifampicin TB (RR-TB) and multi-drug resistant TB (MDR-TB), was deemed as the gravest crisis, and the estimated cases in China accounted for 13% of global totals, also ranked second. Thus, realizing the target of End TB by the control and prevention of TB epidemics has become urgent in China.

In China, there are a variety of epidemic characteristics due to different influential factors like socio-economic indices and diverse regions^3^. Zhejiang Province is a developed economy in the eastern region of China^4^. Although decreased TB incidence in this region was reported, the current velocity of reduction may not be adequate for reaching future demands of WHO’s End TB strategy and the UN Sustainable Development Goals’ target^5^. During the recent decade, Zhejiang Province reported nearly 300,000 notified cases. Thus, the exploration of the potential implications hidden in this information was essential. Based on previous studies, the time series model could be used to carry out short-term predictions effectively, providing useful clues and evidence for the control and prevention of TB in the future^6,7^. Among several time series models, the autoregressive integrated moving average (ARIMA) model, including the seasonal ARIMA model, takes several key variables into account including periodic variables, random factors, and actual fluctuation caused by epidemics^8,9^. The model also has distinct advantages like the requirement of limited data variables and high prediction accuracy^10^. ARIMA model has been widely used in the field of infectious diseases like hemorrhagic fever with renal syndrome (HFRS), hand foot and mouth disease (HFMD), avian influenza, and TB^11–15^.

Thus, this study aimed to explore the underlying burden of TB in the past ten years, find the regulation of TB among different genders, discern the target groups, and predict further epidemics in Zhejiang Province. This study might not just contribute to the advancement of further health policy for TB control at the regional level but also provide useful references for TB prevention in China.

## Materials and Methods

### Study area

**Figure 1:**
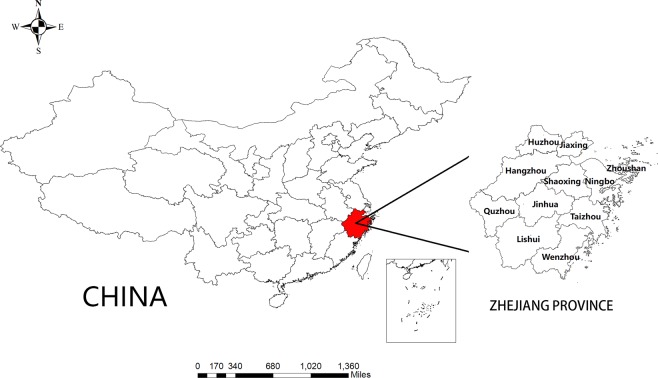
Area of Zhejiang Province in China.

Zhejiang Province is in the eastern region of China with a land area of nearly 101,800 square kilometers, accounting for 1.06% of China^7^. As an economically developed province with a GDP of 6 trillion RMB in 2019, it consists of 11 regional cities: Hangzhou, Ningbo, Wenzhou, Jiaxing, Huzhou, Shaoxing, Jinhua, Quzhou, Zhoushan, Taizhou, and Lishui. As the smallest province in China, it has two sub-provincial cities and is composed of nearly 90 counties. In 2018, there was a reported total of 57.37 million permanent people and a migrant population of approximately 26 million in Zhejiang Province, which contributed to the complexity in controlling and preventing TB^4^. The location of the Zhejiang province is shown in Fig. 1.

### Data collection

All included data was collected by date of notification from the Web-based TB Information Management System (TBIMS) in China, which was established in 2005^16^. In this system, all notified TB cases including new and relapse cases were recorded in the designated hospital at the levels of county, city and province then checked by the local Centers for Disease Control and Prevention (CDC) in Zhejiang Province. In this study, the details of TB cases including gender, year, date of notification, reported city, etc. were acquired and analyzed. The residential populations of both genders in Zhejiang Province and other sociodemographic information were obtained from the Chinese Information System for Disease Control and Prevention (CISDCP) and the Zhejiang Statistical Yearbook (free access from official website: http://tjj.zj.gov.cn/col/col1525563/index.html). Permissions of data access in CISDCP and TBIMS were approved by the Zhejiang Provincial Center for Disease Control and Prevention. In this study, some private information, such as patient name, identification number, address, and contact information were excluded. The data was checked and screened by two independents, respectively.

### Case definition

Notified TB cases included in the TBIMS consisted of laboratory confirmed pulmonary tuberculosis (PTB), clinical diagnostic PTB, and extrapulmonary tuberculosis (EPTB). All TB cases were classified based on the National Diagnostic Criteria for Pulmonary Tuberculosis (WS288–2008, WS196-2001, and WS 288-2017) and Classification of Tuberculosis (WS196-2017)^17–19^. The confirmed PTB cases were denoted as people with possible PTB symptoms such as continuous cough for more than two weeks, hemoptysis, night sweat, etc. and confirmed by sputum smear and/or sputum culture with the result of detectable acid-fast bacilli or positive result from a rapid molecular diagnostic instrument (e.g., GeneXpert). Clinical diagnosis of PTB was defined as people with obviously abnormal chest radiography along with no curative effect from anti-inflammatory treatment under the circumstance of negative results from laboratory tests or related result absence^20,21^. EPTB was defined as people who were diagnosed as tuberculosis in other organs other than lung^19^.

### Epidemiological characteristics of TB in Zhejiang Province from 2009–2018

The data were downloaded from the TBIMS. After the data cleaning in our study group, the distribution and epidemiological characteristics of notified TB were presented by year, age, gender, ethnics, occupation, season, reported region, and treatment type, respectively.

### Stratified analysis by gender and rate difference (RD) before-after five years

Using the registered permanent population of both genders in Zhejiang Province, all included cases were categorized into 18 age groups, each consisting of 5 years. The notified incidences of TB in each group were calculated. We used the RD to examine alterations in notified TB incidence by comparing each group with its corresponding senior group. This was defined as the group five years into the future by age and years. If the RD value was positive, it implied a still rising risk in this age-specific group with the definition of positive RD. Otherwise, the RD value hinted at declined risk, denoting negative RD.

### Time-series analysis of ARIMA model

The ARIMA model was first presented by Box & Jenkins in 1970 and consisted of three sections in the order of autoregression (p), the degree of difference (d), and the order of moving average (q)^22^. For seasonal trends, the model presented as ARIMA (p, d, q) × (P, D, Q) s, in which P denoted seasonal autoregression, D as the seasonal differencing degree, and Q as the seasonal moving average. Given the underlying seasonal feature of notified TB, the seasonal model was selected and performed in this study. As is common, the stationarity of data was tested in the first step; if the data was not stationary, the appropriate differencing and/or exponential transformation was conducted to convert the data into a stationary series. In addition, autocorrelation function (ACF) and the partial autocorrelation function (PACF) were used to identify the q and p^23^. Ljung-Box tests were also used to perform the white noise test, and indicators like Akaike’s Information Criterion (AIC) and Bayesian Information Criterion (BIC) were adopted to screen the optimal model^24^.

### Ethics statement

This research was approved by the Ethics Committee of the Zhejiang Provincial Center for Disease Control and Prevention. All personal information in this study was kept confidential as required.

### Statistical analysis

The descriptive analysis and Mann-Kendall test were performed by R software (version 3.5.3) and Microsoft Excel, and the map presentation used the ArcGIS software (version 10.2, SERI Inc.; Redlands, CA, USA). The time series model was determined using R software (TSstudio package). All results were considered statistically significant at *P* < 0.05 with two sides.

## Results

### General epidemiological characteristics of TB

**Figure 2:**
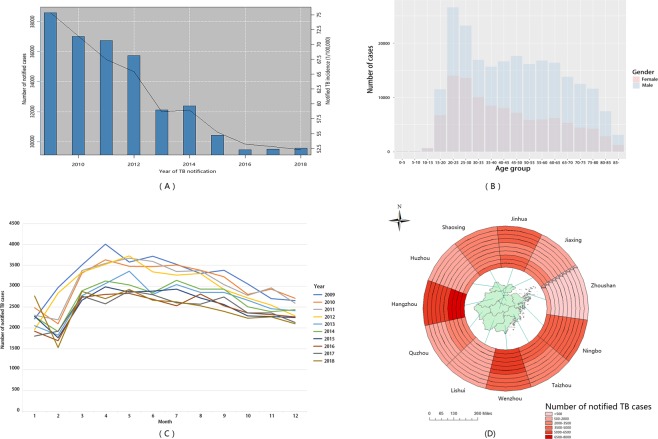
General Epidemiological Characteristics of TB in Zhejiang Province between the Period 2009–2018. (**A**) Number of notified TB cases in Zhejiang Province during the period 2009–2018; (**B**) Number of notified TB cases for both genders among different age groups; (**C**) The seasonal distribution of notified TB cases; (**D**) The regional distribution of notified TB cases in Zhejiang Province. These were created by R software (3.5.3), Excel (Microsoft Excel 2016), and ArcGIS software (version 10.2, ESRI Inc.; Redlands, CA, USA). URL http://www.R-project.org; https://www.esri.com/.

From 2009 to 2018, there was a total of 331,594 notified TB cases in the national surveillance system from Zhejiang Province, with a gender ratio of male to female 2.16:1. The number of males in all age groups was more than females. The top 10 ethnic groups were listed and the Han ethnic group accounted for more than 96% of notified TB cases. Also, a declining trend with significance by year was identified in Han, She and Mongolia ethnic groups, respectively (Supplement Table 1, determined by Mann-Kendall test). The sum of peasants and workers accounted for nearly 70% of notified cases in the study period. Additionally, the TB notification incidences showed a declining trend from 75.38/100,000 in 2009 to 52.25/100,000 in 2018. The nadir of notified TB cases was identified in February, and then the number of cases reached a peak in April with a persistent decline in the following months. For the regional distribution, Hangzhou, Ningbo, Wenzhou, and Jinhua had more TB cases than other cities, although recent decades witnessed decreasing case numbers in each prefecture. These are shown in Fig. 2. Furthermore, our results showed that the proportion of relapse cases accounted for nearly 7% of all notification TB cases in the study period (Supplement Table 2).

### The stratified analysis of notified TB by age groups in males and females

To find the accurate notified TB incidence in each age-specific group, the permanent population during the study period was used to standardize notified TB incidence. From Tables 1 and 2, the notification rate of different genders in each age group nearly all showed a declining trend, particularly in the groups of “20–50” and “>75”. Additionally, all age groups under 15 demonstrated a lower notified TB incidence in both male and female populations. Notified TB incidence rose sharply and reached its first peak within the age brackets of “15–35” in males and females. Following, the rate showed a slight decline and arrived at the second peak after “>55” for both genders. For the second peak, the incidence for males was higher than the female group by nearly 3–4 times. The details are shown in Tables 1 and 2.

**Table 1:** Standardized Notified TB Incidence (1/100,000) in Males from 2009 to 2018.

Age groups	Year									
	2009	2010	2011	2012	2013	2014	2015	2016	2017	2018
0-	1.87	1.49	0.89	0.78	0.55	0.84	0.75	0.22	0.37	0.79
5-	1.34	1.01	1.78	1.46	0.6	1.25	0.26	0.81	0.47	0.53
10-	3.56	4.25	4.59	5.92	4.73	3.62	5.19	5.33	4.87	7.38
15-	69.78	69.01	62.77	72.77	75.67	68.34	63.8	66.15	57.97	51.96
20-	167.55	172.27	166.75	122.13	105.33	98.75	83.56	88.94	83.08	78.2
25-	156.39	150.19	131.05	108.11	97.47	98.14	93.3	95.28	89.71	86.72
30-	128.41	118.35	102.42	81.81	76.24	70.6	64.54	68.58	68.52	68.7
35-	96.75	97.25	81.83	70.79	62.06	59.04	52.31	48.53	46.85	48.06
40-	88.93	84.88	76.92	72.01	60.87	63.42	53.57	50.59	49.61	46.37
45-	101.55	89.54	84.53	75.38	59.91	62.31	56.42	53.53	53.8	52.75
50-	87.83	76.13	65.15	84.76	81.69	84.79	82.91	86.6	88.7	90.88
55-	107.74	96.76	86.18	108.46	99.06	93.49	85.71	76.96	67.65	79.94
60-	141.29	121.09	103.66	122.53	109.58	107.06	105.48	104.48	116.9	120.59
65-	191.14	157.61	139.53	167.4	160.04	158.09	166.66	146.3	156.67	140.61
70-	239.97	218.54	167.21	191.05	156.94	175.58	169.62	142.83	163.5	156.43
75-	303.78	276.29	225.56	210.55	200.09	194.77	175.77	146.54	137.21	115.38
80-	299.49	284.43	236.66	211.83	207.66	217.46	220.05	176.56	191.65	175.42
>85	398.15	370.47	337.9	163.39	167.94	153.51	200.05	176.42	193.19	172.44

**Table 2:** Standardized Notified TB Incidence (1/100,000) in Females from 2009 to 2018.

Age groups	Year									
	2009	2010	2011	2012	2013	2014	2015	2016	2017	2018
0-	0.94	0.74	1.17	0.63	0.73	0.36	0.27	0.26	0.26	0.17
5-	1.03	1.09	1.29	0.82	0.74	0.52	0.68	0.6	0.6	0.59
10-	5.99	5.41	5.62	6.66	5.37	4.61	6.83	8.84	5.87	6.03
15-	49.78	53.15	46.86	41.85	42.07	47.83	39.55	35.69	36.89	31.92
20-	103.51	105.63	97.17	70.93	60.34	57.02	48.68	44.84	43.3	41.04
25-	88.66	88.41	81.39	68.61	61.03	61.4	61.9	59.22	56.78	50.61
30-	65.03	67.33	58.85	50.27	50.81	47.1	45.42	44.4	45.67	46.54
35-	49.47	51.57	44.37	38.03	33.56	34.74	30.64	29.56	26.54	30.57
40-	42.15	39.81	37.06	36.82	29.14	32.15	26.52	27.48	24	26.04
45-	39.8	35.25	36.32	33.25	23.59	26.82	24.27	24.13	23.73	22.84
50-	30.17	26	23.83	30.45	32.66	33.99	35.63	34.8	38.29	38.36
55-	43.61	34.58	32.06	43.02	33.31	33.65	30.19	27.8	25.5	28.56
60-	50.83	49.06	41.76	49.22	41.59	43.22	43.67	41.38	43.18	45.8
65-	67.29	64.51	50.79	68.53	66.83	68.81	68.85	63.43	64.52	54.64
70-	68.66	70.54	60.95	68.61	66.66	68.25	68.54	66.71	64.77	64.03
75-	85.72	82.42	72.02	70.63	68.49	74.4	68.96	53.29	52.97	47.26
80-	74.4	67.86	70.19	67.65	67.72	81.58	72.54	63.05	69.53	57.92
>85	75.27	87.63	69.35	42.53	50.87	52.01	56.79	49.55	48.77	49.21

### Rate difference (RD) of notified TB before-after five years among the study population

**Figure 3:**
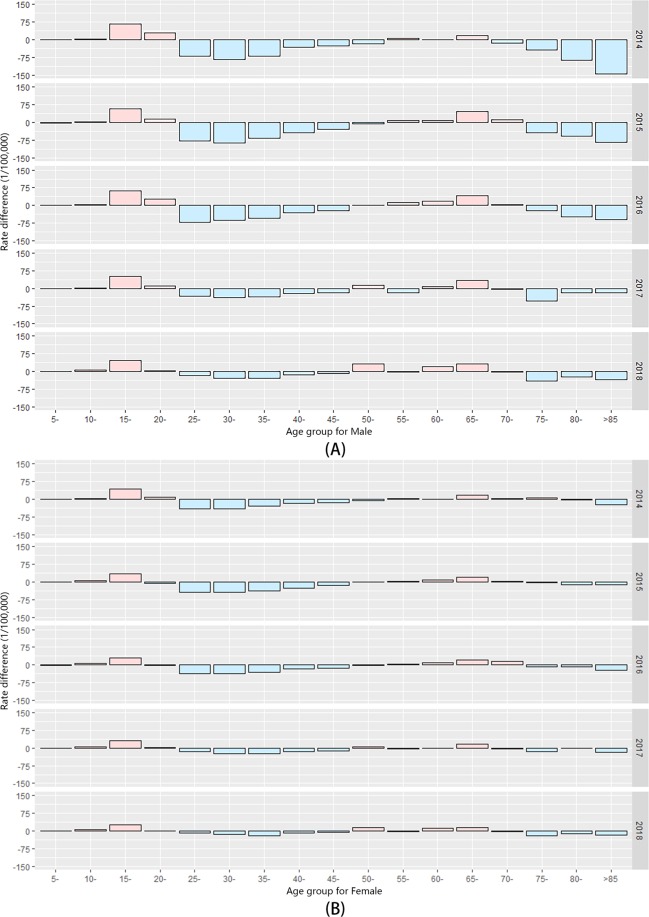
RD of Notified TB Incidence Before-After Five Years in the Study Period. The RD in different age groups of males (**A**) and female (**B**); the pink histogram above the horizon represents increased incidence, while the light blue histogram under the horizon indicates the decreased incidence of TB. These were created by R software (3.5.3). URL http://www.R-project.org.

In this study, all notified TB cases were classified into five-year age groups. We considered age groups in 2014–2018 to be the follow-up of age groups younger by five years in 2009–2013. For these five comparison groups, the total trend of RD in each year (2014–2018) was nearly the same. For males, the positive RD of notified TB incidence focused on the age groups of “5–25” and “50–70”, which was similar to females. The age groups of “25–50” and “>75” presented negative RD of notified TB incidence while the differences were diminishing in both genders. These results are shown in Fig. 3.

### Time-series analysis of notified TB cases

**Figure 4:**
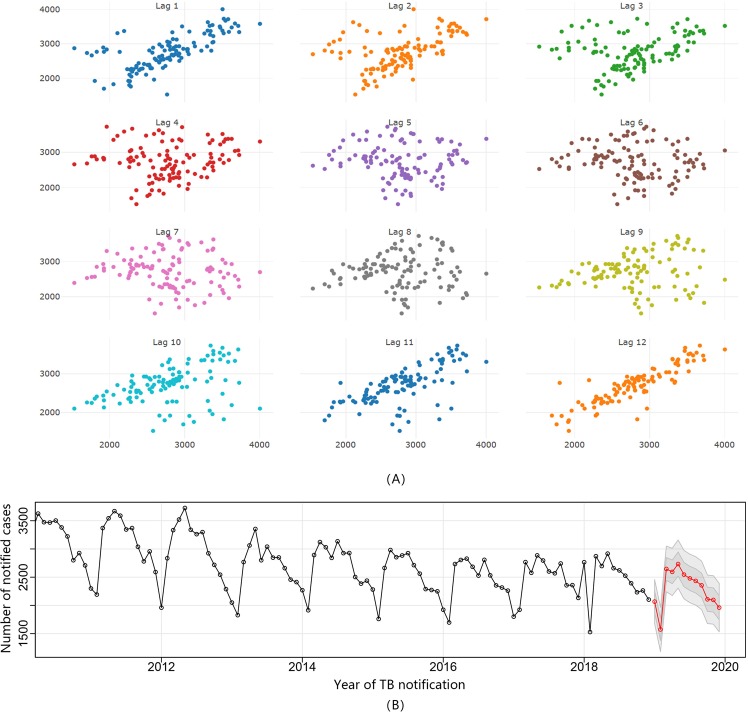
Periodic Identification of Notified TB Cases and Trend Prediction of TB in Zhejiang Province. (**A**) The identification of potential periodicity of TB notification. With the change of different lag, the scatter diagram showed an obvious linear trend at lag 12, implying a potential periodicity of 12 months; (**B**) Number of notified TB cases predicted by the SARIMA model. The TBIMS data in the study period was used as the training dataset to construct the model and predict notified TB cases with an 80% confidence interval (CI) and 95% CI in 2019. These figures  were created by R software (3.5.3). URL http://www.R-project.org.

In this study, we used R software to identify the predicted model of notified TB cases. Given the obvious periodic feature of TB occurrence, the seasonal ARIMA model was constructed in this study. Ultimately, SARIMA (2,0,2) (0,1,1)12 (AIC = 1465.9, BIC = 1484.7) was determined as the optimal one. The further prediction of notified TB cases in 2019 is shown in Fig. 4 and Table 3. Additionally, the estimated parameters for this SARIMA model are presented in Table 4.

**Table 3:** Predicted Notified TB Cases by SARIMA (2,0,2) (0,1,1)12 and the Actual Notified Number in 2019.

Time	Point estimation	The upper value of 95% CI	The lower value of 95% CI	The notification number
Jan-19	2068	1674	2461	2154
Feb-19	1574	1179	1969	1626
Mar-19	2646	2239	3054	2628
Apr-19	2594	2172	3016	2707
May-19	2733	2304	3163	2662
Jun-19	2546	2114	2978	2397
Jul-19	2479	2046	2911	2620
Aug-19	2434	2001	2867	2428
Sep-19	2356	1923	2789	2292
Oct-19	2108	1675	2541	2110
Nov-19	2098	1665	2530	1942
Dec-19	1961	1529	2394	1950

**Table 4:** Estimated Parameters of SARIMA for the Prediction of Notified TB Cases.

Parameter*	RMSE	MAE	MPE	MAPE	MASE
Value	185.17	134.78	−0.28	5.17	0.75

*RMSE: root mean square error; MAE: mean absolute error; MPE: mean percentage error; MAPE: mean absolute percentage error; MASE: mean absolute square error.

## Discussion

Although TB is deemed as a preventable and treatable disease, it still causes a considerable global disease burden^25^. According to the release of the Global Burden of Disease (GBD) in 2016, the annualized age-standardized change in TB incidence among HIV-negative individuals was −1.3% [−1.5 to −1.2] and −4.0% [−4.5 to −3.7] in HIV-positive individuals, rates that would not meet the demand of the End TB strategy by 2030^25^. Additional analysis of China in GBD 2015 demonstrated a slow decline in TB mortality and DALYs in recent years^26^. That is to say, some existing prevention, control strategies, and policies should still be improved. Zhejiang Province, as a developed area in China with a GDP like the Netherlands, recently undertook numerous explorations and endeavors to accelerate the realization of the End TB goal such as improving etiology diagnosis by popularizing Gene-Xpert technology, promoting treatment compliance through the implementation of electronic pillbox, and implementing health insurance reform for TB patients through payment reform, etc. Also, International Cooperation Projects like the Global Fund TB Program and China-Bill Melinda Gate Phase III provided a new horizon to control TB epidemics in local regions. Thus, the identification and exploration of regulation in the past decade was of importance in summarizing the previous practices, identifying existing insufficiencies, and providing advice for optimizing available health policy.

In this study, we included nearly 332,000 notified TB cases in the recent decade. In total, the incidence of notified TB declined by about 30%, which was inseparable with the actions above in Zhejiang Province. However, there was an obvious higher proportion of cases in the male population. According to the annual monitoring report in China, the average proportion of male to female in the whole population was about 2.19:1, which was similar to the proportion in Zhejiang Province. In different countries and regions, the ratio of male to female also demonstrated disparities. Previous TB prevalence surveys demonstrated a ratio of 1.2 in Ethiopia and 4.5 in Vietnam^27,28^. Given the average sex ratio of 1.9:1 around the globe, this implies a higher burden in the male population in China and Zhejiang Province^29^. This appearance has also presented in some low-burden countries^30,31^. Interestingly, one study in Germany demonstrated that the prevalence of latent TB infection had no difference in gender distribution while active TB in males showed an apparent dominance^32^. Thus, further study should be considered in the field of immune responses and inflammatory responses to find its potential mechanism^28^; meanwhile, more specific public health interventions, community health education and policy support should be considered to lower TB transmission^20^. The Han ethnic group accounted for the majority in Zhejiang Province, and the continuous decline of notified TB cases in this group was consistent with the trend of our findings in the whole population. Similar to the previous findings, the peasants and workers were the commonly vulnerable population for TB occurrence in Zhejiang Province, implying these occupation populations should still be prioritized in the developed area^33^.

The phenomenon of declined notification cases in January and February accompanied with the rapid rise around March and April was attributable to two factors. The first one, the Spring Festival, also known as the Lunar New Year in China, occurred in January or February. More patients seek medical care after this grand festival. Due to the concentration of family gatherings in the spring festival, delayed medical presentation might further aggravate potential TB infection and transmission. Therefore, more health education should be carried out before this period. Furthermore, health checkups for college enrollment examinations around March and April might also contribute to the increased identification of active TB cases with no/mild symptoms.

In spatial distribution, more notified cases were reported in Hangzhou, Ningbo and Wenzhou city, which were ascribed to their large population in some part, but also associated with the developed economic level that attracted more migrant populations in other regions of Zhejiang Province and outside the province^33^. Therefore, combined with a previous study, more holistic policies such as comprehensive health-care policy should be put forward to give full coverage to all people in the community, which could reduce the risk of treatment interruption in patient groups, particularly in migrant groups with low socio-economic conditions^34^.

The stratified analysis was performed in both gender populations. Broadly speaking, the notified TB incidence in males was higher than that in females. For both genders, the low occurrences were concentrated in ages under 15, which might be attributable to the efficacy of Bacillus Calmette-Guerin (BCG) in protection against childhood and disseminated TB^35^. This finding also proved to some extent that the preventive effect of BCG might cover the first decade of life, which was consistent with previous long-term results^36^. For both genders, age groups from “15–35” and “>55” showed a high notification incidence, especially in the senior age group, while the trend for females was relatively flat. The rising incidence in the “15–35” age group might be correlated with the attenuation of protective efficacy and the increased exposure of environmental mycobacteria that reduced reactivity to BCG^37–40^. Thus, for the student population during the age period of “15–35”, it is suggested that the school infirmary provide additional care to students with TB symptoms, especially for students residing in the same dormitory, which might imply the possibility of TB clustering. For manual workers, workplaces should provide regular physical examinations and necessary promotion and education to enhance early TB findings and reduce clustered epidemics. For the age group of “>55”, the increased TB incidence might be attributed to the low immunity in this specific population, particularly in the diabetic population with limited control levels of blood glucose^20^. Our previous study also demonstrated that active case findings could decrease the active TB incidence in some target populations^20^. In the future, more elaborated and comprehensive actions should be formulated and explored to reverse this TB epidemic in Zhejiang Province.

RD of notified TB incidence before-after five years were analyzed in our study. To our knowledge, the notification rate of TB in each age group might be influenced by several factors such as internal immune levels and existing supervision (like underdiagnosis and underreporting, etc.) combined with corresponding external health policy (medical insurance and special government subsidies, etc.)^16,41,42^. Thus, we used the RD to offset some internal effects such as the efficacy of BCG and identified the underlying external reasons, providing evidence for strengthening health strategies and advancing health policies. Based on the available results, although the overall notified TB incidence experienced a decline, we still found increased notification of TB among age groups “15–20” and “65–70” in both genders. For the age group of “15–20”, the majority was a student population around the period of high school to college and the relative risk ranged from 19.2 to 10.9 from 2014 to 2018^43^. Despite a successive decline of TB risk in this specific group, the rate of descent was insufficient under the existing health policy. Given that the only uniform medical checkup for students occurs during college enrollment, we appealed to have routine checkups conducted annually for students in this age group to enhance TB identification and integrate this strategy into further policies of TB control in Zhejiang Province. For the age group of “65–70”, the increased TB notification incidence was attributable to recent efforts in health examination for the elderly, which improved the finding of active TB in this age group and correlated with the further decline in the older age group^20,44,45^. Thus, it is suggested that more comprehensive physical examination involving TB identification such as GeneXpert should be considered in some developed areas with a high TB incidence. Moreover, other age groups demonstrated declining notified TB incidence, implying the effectiveness of current health control and prevention strategies. Yet, the diminishing negative value of RD and absolutely high incidence in these groups might illustrate that novel implementations and strategy combined with the integration of early identification, clinical treatment and community management for TB control should be explored.

Ultimately, the SARIMA (2,0,2) (0,1,1)12 model was chosen and applied to the prediction of notified TB cases in Zhejiang Province. Comparing our predictions with actual notified TB number from the TBIMS system in 2019 demonstrated the accuracy in our model’s fit. Previous studies in other regions also used the SARIMA model to give a short-term prediction of TB epidemics with high predictive precision, which was consistent with our findings^46,47^.

## Limitations

Some limitations should be listed in this study. Firstly, the data we used was notification records. Due to differences in notification quality amid different regions, some bias in the results might be unavoidable. Due to the paucity of details involving socio-economic parameters, TB latent infection data, and drug-resistance information throughout the study period, we did not analyze these factors in our study, which might not reveal the comprehensiveness of TB occurrence. Besides this, although we had prudently drawn some conclusions, we did not take the potential influence of the migrant population from other provinces into account, which might also have had an effect in our available results. In addition, we tried to explore the possible epidemiological characteristics of TB notification in eastern China while the data from one province might not give a full description. Finally, the model we had chosen was a common one with the possibility of overfitting, and another more suitable integrated model such as the ARIMA-NAR hybrid model was not considered in this study.

## Conclusion

In general, the notification of TB incidence in Zhejiang Province was declining in the past decade while the male population and critical months from January to April still need special attention. Some implementations such as the increased frequency of checkups in specific student groups and strengthening of elder health examination could be explored and integrated into available health policy. Ultimately, the SARIMA model can be used to fit trends in TB notification cases well, and can be used in the further prediction of TB in Zhejiang Province.

## Supplementary information

Supplementary information.
