# Supplementary material for: Identification and Prediction of Tuberculosis in Eastern China: Analyses from 10-year Population-based Notification Data in Zhejiang Province, China
Source: Sci Rep. 2020 May 4;10:7425. doi: 10.1038/s41598-020-64387-5 (PMC7198485; doi:10.1038/s41598-020-64387-5)
Supplement: Supplementary file 1 — Supplementary information. [file 41598_2020_64387_MOESM1_ESM.docx]

**Identification and Prediction of Tuberculosis in Eastern China: Analyses from 10-year Population-based Notification Data in Zhejiang Province, China**

Kui Liu, Tao li, Avina Vongpradith, Ying Peng, Yu Zhang, Fei Wang, Wei Wang, Chengliang Chai, Songhua Chen, Lin Zhou, Xinyi Chen, Qiao Bian, Bin Chen, Xiaomeng Wang, Jianmin Jiang

**Supplement Table 1. The Trend of Notified TB Cases in Various** **Ethnic Groups during the Study Period.**

|  | Year | | | | | | | | | | Z value* | *P* value |
| --- | --- | --- | --- | --- | --- | --- | --- | --- | --- | --- | --- | --- |
|  | 2009 | 2010 | 2011 | 2012 | 2013 | 2014 | 2015 | 2016 | 2017 | 2018 |  |  |
| **Ethnic Group** | | | | | | | | | | | | |
| Han | 37947 | 36059 | 35522 | 34688 | 31059 | 31199 | 29525 | 28435 | 28708 | 28872 | -3.22 | *P*<0.05 |
| Miao | 142 | 209 | 302 | 232 | 234 | 288 | 216 | 244 | 201 | 148 | -0.18 | 0.86 |
| Tujia | 111 | 168 | 221 | 197 | 176 | 211 | 136 | 192 | 119 | 111 | -0.81 | 0.42 |
| Yi | 59 | 98 | 126 | 125 | 134 | 159 | 164 | 147 | 94 | 105 | 1.07 | 0.28 |
| Bouyei | 64 | 125 | 152 | 146 | 153 | 128 | 109 | 128 | 117 | 88 | -0.63 | 0.53 |
| Dong | 23 | 54 | 77 | 61 | 66 | 81 | 41 | 51 | 32 | 32 | -0.63 | 0.53 |
| She | 63 | 64 | 42 | 42 | 41 | 46 | 24 | 43 | 23 | 36 | -2.07 | *P*<0.05 |
| Zhuang | 26 | 31 | 47 | 44 | 38 | 58 | 41 | 42 | 45 | 37 | 0.72 | 0.47 |
| Mongolia | 47 | 61 | 56 | 39 | 30 | 38 | 31 | 23 | 31 | 29 | -2.60 | *P*<0.05 |
| Hui | 20 | 19 | 23 | 16 | 31 | 20 | 20 | 25 | 23 | 26 | 1.46 | 0.14 |
| Others | 94 | 118 | 176 | 157 | 164 | 158 | 127 | 139 | 117 | 92 | -0.89 | 0.37 |

*: Mann-Kendall test.

**Supplement Table 2****. The Proportion of New Cases and Relapse Cases in the Study Period.**

| Year | | | | | | | | | | | |
| --- | --- | --- | --- | --- | --- | --- | --- | --- | --- | --- | --- |
|  | 2009 | 2010 | 2011 | 2012 | 2013 | 2014 | 2015 | 2016 | 2017 | 2018 | Total |
| **Classification of Registration** |  |  |  |  |  |  |  |  |  |  |  |
| Newly cases (%) | 34890 | 33641 | 33598 | 32501 | 29229 | 29240 | 27648 | 26849 | 26757 | 27019 | 301372 |
|  | (90.40%) | (90.91%) | (91.44%) | (90.92%) | (90.98%) | (90.29%) | (90.85%) | (91.11%) | (90.67%) | (91.35%) | (90.89%) |
| Relapse cases (%) | 2724 | 2618 | 2477 | 2580 | 2365 | 2452 | 2091 | 2084 | 2176 | 2184 | 23751 |
|  | (7.06%) | (7.07%) | (6.74%) | (7.22%) | (7.36%) | (7.57%) | (6.87%) | (7.07%) | (7.37%) | (7.38%) | (7.16%) |
| Others (%) | 982 | 747 | 669 | 666 | 535 | 711 | 695 | 536 | 577 | 372 | 6490 |
|  | (2.54%) | (2.02%) | (1.82%) | (1.86%) | (1.67%) | (2.20%) | (2.28%) | (1.82%) | (1.96%) | (1.26%) | (1.96%) |
